# Genesis of a CO_2_-rich and H_2_O-depleted atmosphere from Earth’s early global magma ocean

**DOI:** 10.1126/sciadv.abj0406

**Published:** 2021-10-06

**Authors:** Natalia V. Solomatova, Razvan Caracas

**Affiliations:** 1 CNRS, Ecole Normale Supérieure de Lyon, Laboratoire de Géologie de Lyon LGLTPE UMR5276, Centre Blaise Pascal, 46 allée d’Italie, Lyon 69364, France.; 2 The Center for Earth Evolution and Dynamics (CEED), University of Oslo, Blindern, Oslo, Norway.

## Abstract

The magma ocean was a important reservoir for Earth’s primary volatiles. Understanding the volatile fluxes between the early atmosphere and the magma ocean is fundamental for quantifying the volatile budget of our planet. Here we investigate the vaporization of carbon and hydrogen at the boundary between the magma ocean and the thick, hot early atmosphere using first-principles molecular dynamics calculations. We find that carbon is rapidly devolatilized, while hydrogen mostly remains dissolved in the magma during the existence of a thick silicate-bearing atmosphere. In the early stages of the magma ocean, the atmosphere would have contained significantly more carbon than hydrogen, and the high concentrations of carbon dioxide would have prolonged the cooling time of early Earth.

## Teaser

Under a thick atmosphere, carbon is rapidly vaporized from the magma ocean, while hydrogen remains dissolved in the silicate melt.

## INTRODUCTION

Quantifying the total amount of volatiles stored in hidden reservoirs of Earth is crucial for our understanding of the global volatile cycle. To estimate the volatile content of Earth’s interior, we must gain a better understanding of their fluxes throughout the history of our planet, starting with the global magma ocean stage (*1*). According to the Giant Impact theory, a planetesimal collided with proto-Earth, melting and partially vaporizing the two bodies to create a disk from which present-day Earth and Moon subsequently formed (*2*–*5*). The behavior of volatile elements and the degree of mixing depend on the impact regime. In the canonical model, a Mars-sized planetesimal obliquely collides with proto-Earth with a low impact velocity, creating a disk composed of a silicate melt interior surrounded by a vapor atmosphere. In high-angular momentum simulations, an impactor hits an oblate fast-spinning proto-Earth, resulting in a hot vapor-rich toroidal planetary body, termed the synestia (*6*–*8*). The synestia consists of a turbulent and partially supercritical fluid where the volatiles are well mixed with the more refractory elements. As the temperature decreases, the liquid phase separates from the synestia, condensing into the molten inner planet. Heavier species in the atmosphere then gradually condense into the magma ocean as the temperature continues to decrease.

A significant portion of the volatiles within the oceans and atmosphere of proto-Earth likely survived the Giant Impact (*9*, *10*), being stirred and mixed within the silicate fluid forming the disk or synestia. It has recently been shown that water may not have been lost from the proto-lunar disk by hydrodynamic escape due to the presence of heavier species in the upper part of the disk, which would have limited the diffusion of hydrogen out of the disk (*11*). Moreover, volatiles may have been delivered to Earth by the impactor itself (*12*). After the Giant Impact, most of the volatiles in the protolunar disk or synestia would have been transferred to early Earth (*13*, *14*) where the volatiles created a thick and hot atmosphere, slowing down the cooling rate of Earth’s magma ocean (*15*–*17*). In early Earth, crystals that formed on the surface were denser and so would have sunk just after nucleation (*18*–*21*), prohibiting the formation of a conductive crust, at least during the early stages of the magma ocean. Consequently, volatiles were likely continuously released from the magma ocean to the thick atmosphere via gas-bearing bubbles until Earth was solidified at about 5 Ma (*17*). The detailed thermochemical evolution of Earth-Moon system and the behavior of volatile elements during this process are open questions in the field.

In this study, we aim to characterize the vaporization behavior of carbon and hydrogen from the global magma ocean of early Earth after the Giant Impact, just after the silicate fluid has cooled down from the supercritical state. During the existence of a thick silicate-bearing atmosphere, the pressures at the base of the atmosphere could have reached several kilobars (*22*). As these conditions are highly challenging for equilibrium experiments, we conduct ab initio molecular dynamics simulations on pyrolite melts containing volatiles, such as CO, CO_2_, and H_2_O. The starting pyrolite composition reproduces the bulk silicate Earth composition (*23*) to within 1 weight % (wt %; table S1). Iron is introduced in the pyrolite melt entirely as ferrous oxide (FeO), indirectly establishing an equivalent oxygen fugacity. The addition of more reduced volatile species (e.g., CO) or more oxidized volatile species (e.g., CO_2_ or H_2_O) moves the oxygen fugacity to respectively more reduced or more oxidizing conditions. We consider volatile concentrations of 0.5 to 2.8 wt % C and 0.2 to 1.2 wt % H, typical of carbonaceous chondrites, at densities ranging from 0.95 to 2.7 g/cm^3^ and temperatures of 3000 to 6000 K. We focus on the volatility of carbon and hydrogen as a function of density for pyrolite melt with carbon added as molecular CO or CO_2_ and hydrogen as molecular H_2_O or atomic H. In addition, in a mixture of the two volatiles, we examine the effect of hydrogen on the volatility of carbon (see Methods for compositional details). We monitor the nucleation and chemistry of nanobubbles that form in the shallow parts and at the surface of a magma ocean, in contact with the hot and dense atmosphere.

## RESULTS

### Volatility of carbon

**Fig. 1.**
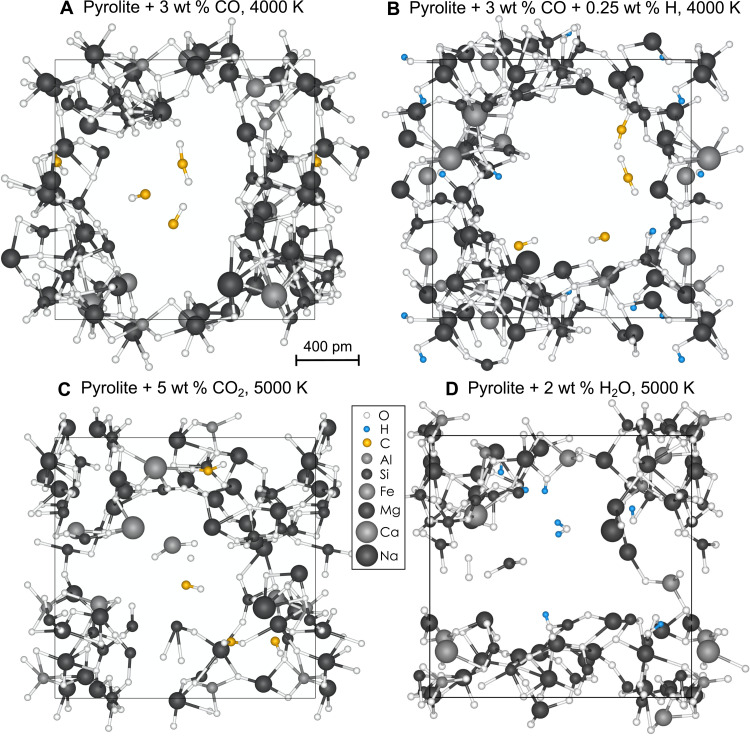
Snapshots of volatile-bearing bubbles in pyrolite melts. (**A**) Pyrolite + 3 wt % CO at 4000 K and 1.1 g/cm^3^ with CO and CO_2_ molecules in the vapor phase. (**B**) Pyrolite + 3 wt % CO + 0.25 wt % H at 4000 K and 1.2 g/cm^3^ with CO and CO_2_ molecules in the vapor phase. (**C**) Pyrolite + 5 wt % CO_2_ at 5000 K and 0.95 g/cm^3^ with CO, O, and FeO_2_ molecules in the vapor phase. (**D**) Pyrolite + 2 wt % H_2_O at 5000 K and 1.4 g/cm^3^ with H_2_O, SiO_2_, and O_2_ molecules in the vapor phase.

At a density of about 2.6 to 2.7 g/cm^3^, similar to the lower-end density range of ultramafic lavas at present-day surface conditions, our simulations predict that carbonated pyrolite melt exists as an entirely polymerized melt without voids or cavities; thus, all of the carbon is dissolved in the silicate melt phase. At 3000 to 6000 K, as the density of the system decreases, nano-sized cavities begin to nucleate spontaneously, which are increasingly populated by volatile species (Fig. 1). The resulting system is a coexistence of pyrolite melt and a vapor-like phase, where the relative proportion of the vapor phase increases with decreasing density. During the simulation, the volatiles continuously exchange between the vapor and melt phase. As the total volume of the cavities grows with decreasing density, the average concentration of carbon in the cavities increases at the expense of the carbon in the silicate melt phase. The ratio between the concentrations of the volatile in the melt and in the gaseous phase yields the volatility and helps us quantify the vaporization of the volatiles as a function of density and temperature.

**Fig. 2.**
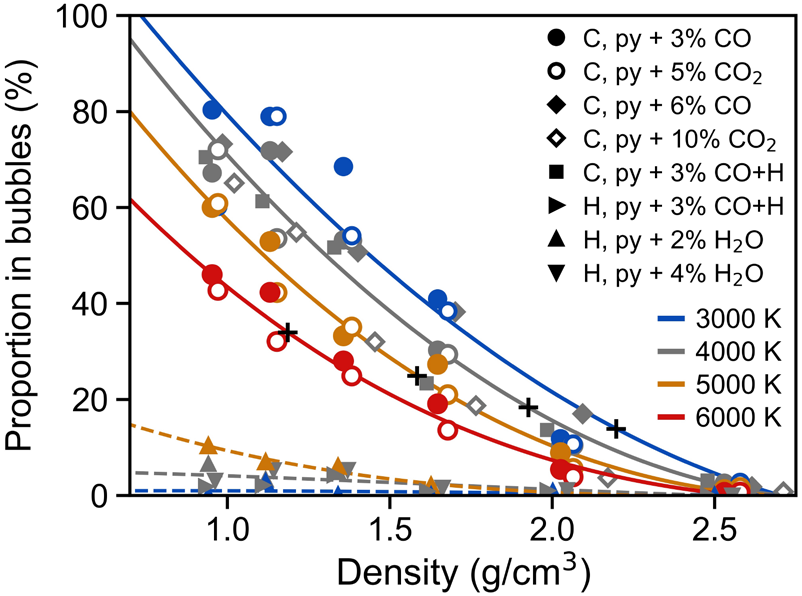
Proportion of carbon and hydrogen in the bubbles relative to the melt as a function of the average melt-vapor system density. No difference was observed in the volatility of carbon for the different compositions. Polynomial fits to the vaporization of carbon and hydrogen are guides for the eyes. There are no error bars on density as the simulations were performed at a defined, constant volume. As a pressure reference, 1 kbar roughly corresponds to a melt-vapor system density of 2.2, 1.9, 1.6, and 1.2 g/cm^3^ at 3000, 4000, 5000, and 6000 K, respectively, marked with black “+” symbols along the carbon vaporization curves. See the main text for more details of the pressure calculations. Pyrolite with 3 wt % CO and 0.25 wt % H is abbreviated as “py + 3 wt % CO+H.”

We compare the volatility of carbon in the more reduced pyrolite melts with 3 to 6 wt % CO and the more oxidized pyrolite melts with 5 to 10 wt % CO_2_. We find that within the resolution of this study, the volatility of carbon is not very sensitive to the oxidation state, the concentration of carbon, or the presence of hydrogen; instead, the volatility of carbon is most strongly dependent on the density (i.e., pressure) and temperature (Fig. 2). Half of the carbon is present in the gaseous phase (i.e., vaporized) at densities of approximately 1.4, 1.3, 1.1, and 0.9 g/cm^3^ and temperatures of 3000, 4000, 5000, and 6000 K, respectively (Fig. 2). Through extrapolation, we predict that all of the carbon would be vaporized by about 0.7, 0.6, 0.5, and 0.2 g/cm^3^ at 3000, 4000, 5000, and 6000 K, respectively. As the carbon travels between the melt and vapor phases, some of the carbon atoms will reside on the interface between the melt and vapor as interfacial carbon. At a density of about 2 g/cm^3^ and the temperatures explored in this study, about 10 to 20% of the carbon exists in the vapor phase, 10 to 20% exists on the interface, and 60 to 80% exists deep in the melt without contact with the bubble surfaces (fig. S1). The amount of carbon on the interface generally remains in the range of about 10 to 30% at all densities and temperatures, decreasing at low densities once most of the carbon becomes vaporized. At 7000 K, the system is above the critical temperature, existing as a supercritical fluid, a single-phase substance with common properties to both a gas and a liquid. Thus, at 7000 K, there are no volatile-bearing bubbles, as there is no coexistence of a gas-like and liquid-like phase.

At ~1500 K, experimental studies estimate that the solubility of carbon in anhydrous mafic melts increases with increasing pressure from about 0.01 wt % C at 1 kbar to 0.1 wt % C at to 5 kbar (*24*, *25*). The effect of temperature on the solubility of carbon is not well constrained within the temperature range of 1000 to 2000 K (*26*) and is unknown for higher temperatures. To determine whether the volatility of carbon would change at much lower carbon concentrations, we perform one simulation with 0.4 wt % C at a system density of 1.3 g/cm^3^ and a temperature of 4000 K. To acquire sufficient statistics despite the low carbon concentration, we performed the simulation for a longer duration that required using significantly more computational resources (see Methods). After integrating over the simulation time, we find that 41% of the carbon exists in the bubbles, slightly lower than the more carbon-rich melts (~50%), but consistent within the scatter of the simulated data. Thus, the volatility of carbon appears to be largely independent of the carbon concentration for the range examined.

**Fig. 3.**
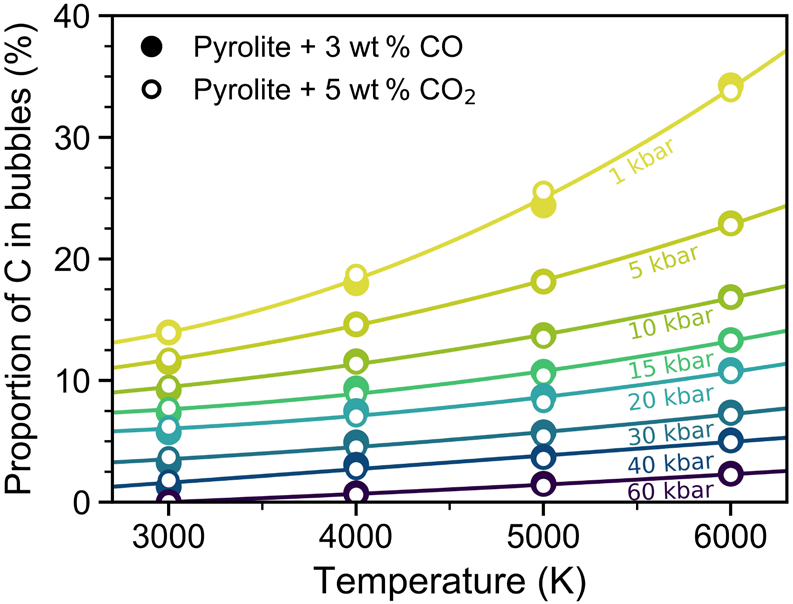
Proportion of carbon in the bubbles as a function of temperature along isobars of 1 to 60 kbar. Pressures were determined from third-order Birch-Murnaghan equations of state at each isotherm (see main text for more details). With increasing temperature and at constant pressure, the proportion of carbon in the bubbles increases with increasing temperature. Polynomial fits are guides for the eyes, demonstrating the increasing sensitivity to temperature with decreasing pressure.

Our simulations are performed at a series of densities along several isotherms. To determine the vaporization behavior of carbon as a function of temperature along isobars, the density must be related to pressure through an equation of state. Although a large fraction of the densities in this study correspond to pressures below 10 kbar, ab initio molecular dynamics simulations are unable to predict low pressures accurately due to the limitation in the number of atoms and the duration time of the simulations (*27*). Thus, we use the results above 1 kbar and fit a third-order Birch-Murnaghan equation of state to determine the pressure-density relations at each temperature, after which we extract carbon volatilization values at selected pressures between 1 and 60 kbar (Fig. 3). This procedure demonstrates the approximate vaporization behavior of carbon along selected isobars. At constant pressure, the volatility of carbon increases with increasing temperature, and the effect of temperature on the vaporization rate of carbon increases with decreasing pressure.

### Speciation of carbon

**Fig. 4.**
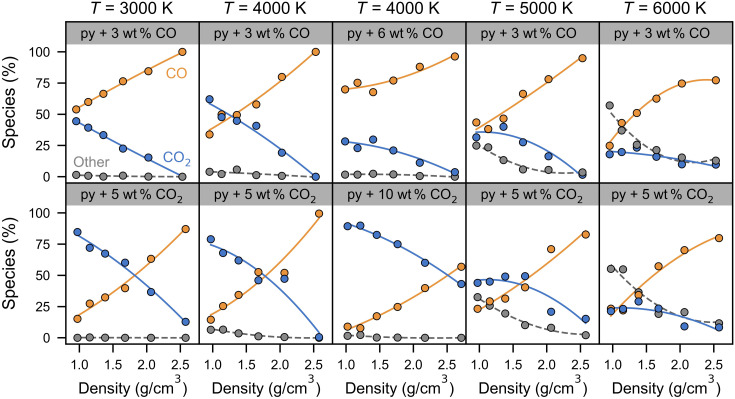
Relative proportion of CO, CO_2_, and other species in the vapor phase as a function of average melt-vapor system density. Each panel shows the amount of CO (orange), CO_2_ (blue), and all other species (gray) in the vapor-bearing bubbles of pyrolite (“py”) melt with 3 to 6 wt % CO and 5 to 10 wt % CO_2_ at 3000 to 6000 K. Species labeled as “other” include vaporized silicate species, such as SiO, SiO_2_, O, and O_2_ (see fig. S5 for their relative abundances in the vapor phase). Second-order polynomial fits are guides for the eyes, showing a general increase in CO concentrations at the expense of CO_2_ with increasing density.

**Fig. 5.**
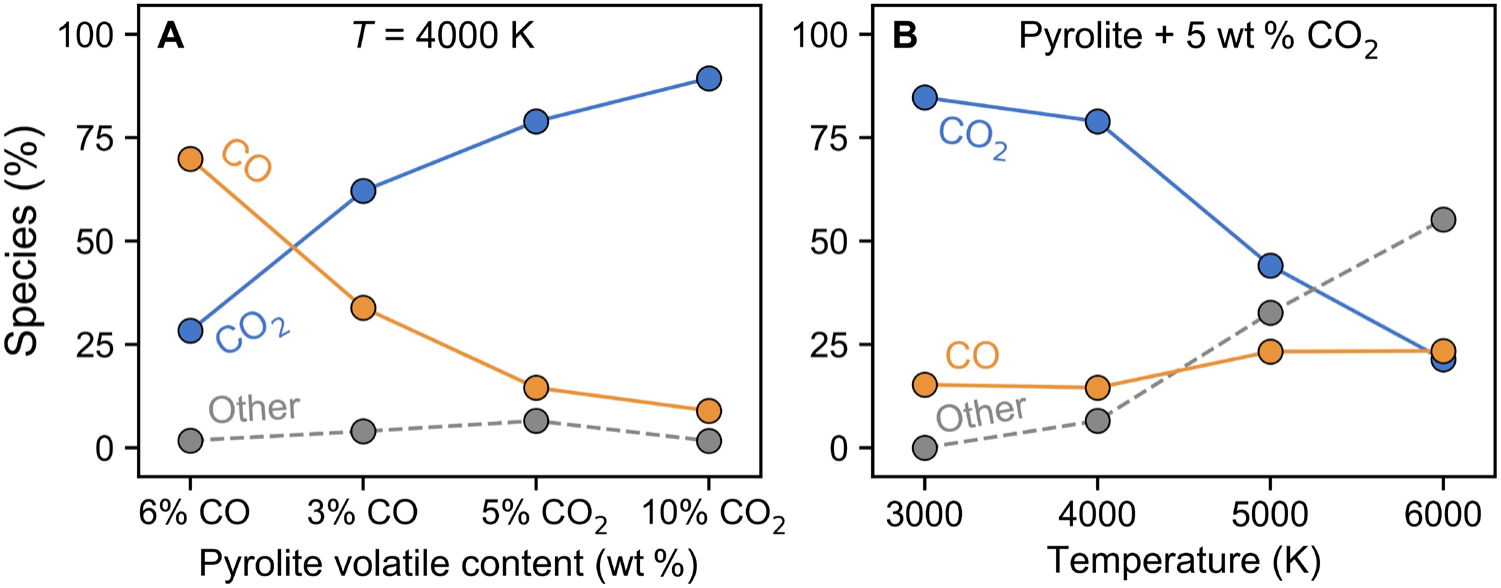
Relative proportion of CO, CO_2_, and other species in the vapor phase as a function of composition and temperature at a melt-vapor system density of ~1 g/cm^3^. (**A**) The effect of pyrolite volatile content on the relative abundance of vaporized species at 4000 K. The pyrolite volatile contents are arranged in order of more reducing to more oxidizing contributions to the pyrolite melt. (**B**) The effect of temperature on the relative abundance of vaporized species for pyrolite with 5 wt % CO_2_. See Fig. 4 for the complete dataset and figs. S2 and S3 for the volatile proportions as a function of volatile content and temperature at additional densities and compositions. Species labeled as other include vaporized silicate species, such as SiO, SiO_2_, O, and O_2_ (see fig. S5 for their relative abundances in the vapor phase).

Although the vaporization rate of carbon does not depend on either the carbon concentration or oxidation state of the melt within the precision of this study, the relative proportions of CO and CO_2_ species found in the bubbles are sensitive to the chemistry of the melt (Fig. 4). Carbon is vaporized almost exclusively as CO and CO_2_, with very rare short-lived species, such as CO_3_ and C_2_O_3_ lasting for 1 to 5 fs, or more exotic species, such as Si_2_CO_4_, existing for several femtoseconds in rare events (i.e., once or twice per simulation length), while atomic carbon was never observed. The bubbles in the more oxidized melt (e.g., pyrolite + 5 wt % CO_2_) have a higher proportion of CO_2_ compared to the bubbles in the more reduced melt (e.g., pyrolite + 3 wt % CO). Thus, the amount of oxygen available in the system affects the speciation of the vaporized carbon, such that the more oxidized system favors more oxidized vapor species. Increasing the amount of molecular species that are introduced in the system (CO versus CO_2_) enhances the amount of that particular molecule in the gas phase, i.e., the bubbles in pyrolite + 6 wt % CO have a higher concentration of CO than the bubbles in pyrolite + 3 wt % CO (Fig. 4). Thus, the amount of oxygen available in the system affects the speciation of the vaporized carbon, such that the more oxidized system favors more oxidized vapor species, while the concentration of CO or CO_2_ units increases the concentration of those respective units in the bubbles (Fig. 5A and fig. S2). That is, the chemistry of the system affects the chemistry of the bubbles, but not the total amount of carbon atoms lost to the bubbles. Conversely, temperature has a strong effect on the total amount of carbon vaporized but has a weaker effect on the chemistry of the vaporized carbon species. The concentration of CO_2_ generally decreases with increasing temperature (Fig. 5B and fig. S3).

With decreasing pressure, the CO_2_/CO ratio in the vapor-filled nanobubbles increases linearly in all the systems explored in this study. Ideal gas calculations on a simple CO_2_-CO-O_2_ gas system predict that with decreasing pressure, CO_2_ should increasingly decompose into CO + 1/2O_2_, as pressure suppresses the thermal dissociation of CO_2_. However, in contact with pyrolite melt, this simple decomposition reaction does not hold anymore. Here, the genesis and speciation of the vapor phase are dictated by the melt composition, as the vaporized volatiles are continuously exchanged between the vapor and melt. In the carbonated pyrolite system, the ratio of CO_2_ to CO in the vapor phase increases with decreasing pressure, likely due to increasingly favorable conditions for losing oxygen from the melt. The CO_2_ molecules in the vapor phase typically exist for 100 to 1000 fs and do not convert to CO + 1/2O_2_ within their lifetime. The concentration of CO_2_ in the vapor also increases with increasing oxygen fugacity and increasing amount of CO_2_ molecules added to the system. Thus, the composition of the melt is a major factor that affects the composition of the vapor phase. After the release of these volatiles into the atmosphere where they are no longer in contact with the magma ocean, their speciation may change.

### Speciation and volatility of hydrogen

Unlike carbon, hydrogen remains predominantly in the melt with less than about 10% of the hydrogen atoms existing in the vapor at all densities and temperatures explored here for pyrolite + 2 to 4 wt % H_2_O (Fig. 2). The low volatility of hydrogen is due to its high solubility in the melt combined with the low oxygen fugacity of the system. Hydrogen begins to vaporize from the melt above at densities below 1.7 g/cm^3^. By about 1 g/cm^3^, the volatility of hydrogen is about 1% at 3000 K, 5% at 4000 K, and 10% at 5000 K. The speciation of hydrogen in the vapor phase appears to become increasingly more H_2_O-rich with decreasing density. At the lowest density examined in this study (~1 g/cm^3^), the speciation of hydrogen in the vapor phase is approximately 70 to 90% H_2_O and 10 to 30% OH^−^ (fig. S4), although the detailed trends with composition and temperature are difficult to determine because of the low occurrences of hydrogen-bearing molecules in the bubbles. Secondary to H_2_O and OH^−^, hydrogen also appears in the bubbles as hydrated silicate vapor species, such as SiOH, FeOH, and NaOH. In the melt, the speciation of hydrogen is almost entirely in the form of OH^−^, attached predominantly to Mg-O*_x_* and Si-O*_x_* polyhedral and, to a lesser extent, as free protons.

At ~1500 K, experimental studies estimate that the solubility of hydrogen in uncarbonated mafic melts increases with increasing pressure from 0.1% H at 0.5 kbar to 0.5 wt % H at 5 kbar (*24*). In this study, we have explored hydrogen concentrations of 0.24 to 0.48 wt % H, which may be below the solubility limit of hydrogen in silicate melts. To examine melts with higher concentrations of hydrogen, we performed simulations on pyrolite melts with 1.1 to 1.2 wt % H (10 to 10.5 wt % H_2_O) at a system density of 1.2 to 1.4 g/cm^3^ where hydrogen was added either as H_2_O or a 50:50 mixture of H_2_O:H_2_, representing more oxidized and more reduced conditions, respectively. In both melts, the volatility of hydrogen remains below 6%. Pursuing higher concentrations is unwarranted, as even wet carbonaceous chondrites rarely reach concentrations above 1 wt % H.

### Volatility of other species

Silicate vapor species are increasingly vaporized with increasing temperature and decreasing density (Fig. 4). At temperatures of 3000 to 4000 K, the vapor phase consists almost entirely of CO and CO_2_ even up to the lowest densities explored where silicate vapor species account for less than 6% of the vapor phase. In contrast, at temperatures of 5000 to 6000 K, a significant fraction of the vapor phase consists of vaporized silicate species, such as SiO, SiO_2_, O_2_, O, Mg, Na, and FeO_2_, in general agreement with the types of species predicted with thermodynamic vaporization models (*28*). At a density of ~1 g/cm^3^, about a third of the species are silicate vapor at 5000 K, and half of the species are silicate vapor by 6000 K. The appearance of vaporized silicate rock species at 5000 K is consistent with previous ab initio and thermodynamic calculations on various silicate melts where O_2_ and SiO vapor species are nearly nonexistent below 4500 K (*29*–*31*). The relative proportions of the four most abundant silicate vapor species (i.e., SiO, SiO_2_, O, and O_2_) are shown in fig. S5 at density-temperature conditions where silicate vapor represents a significant portion of the vapor phase.

We do not observe the formation of methane in the pyrolite melts containing both carbon and hydrogen. The placement of four methane molecules in the void spaces of pyrolite melt at 4000 K results in the complete dissociation all four methane molecules after about 7 ps. Methane is not expected to exist in the vapor phase at the temperatures considered in this study (i.e., 3000 to 6000 K). Chemical equilibrium calculations on the vaporization of various types of chondritic planetesimals at 100 bars show that the concentration of CH_4_ hits a maximum at about 500 to 1000 K and drops off rapidly to trace concentrations in favor of H_2_, H_2_O, CO, and CO_2_ above about 1000 to 1500 K (*32*). Thus, we anticipate that methane would only form after the magma ocean has cooled below ~1500 K.

## DISCUSSION

The magma ocean must have contained a certain amount of volatiles that were retained or acquired after the Giant Impact (*9*–*11*, *13*, *14*). The early stages of the global magma ocean featured temperatures and atmospheric pressures much higher than found anywhere on Earth today. Close to the interface with the atmosphere, temperatures were on the order of several thousands of kelvin and pressures on the order of at least several kilobars (*22*). At these conditions, gas bubbles from the magma transferred part of these volatiles from the deep molten mantle to the hot, thick atmosphere. In this study, we predict the first vapor molecules that were released from the magma ocean.

We find that carbon is rapidly devolatilized, while hydrogen remains mostly dissolved in the silicate melt at pressures corresponding to the shallow parts of a magma ocean under a thick silicate-bearing atmosphere. Although there are no experimental data on bubble nucleation and chemistry at the conditions explored in this study, previous numerical and thermodynamic models have estimated the composition of the gas formed from volcanic melts at elevated pressures (*33*–*35*). Phenomenological numerical models of mid-ocean ridge basalt (MORB) melts at 1473 K estimate that carbon-bearing bubbles nucleate at a depth of about 48 km with the concentration of carbon in the bubbles increasing sharply from 0 to 35% within a 2-km range from a depth of 48 to 46 km due to the supersaturation required for macroscopic bubble nucleation (*33*). Above a depth of 46 km, the numerical models predict an almost linear increase to 95% vaporization at 2 km in depth. Meanwhile, hydrogen is mostly retained in the melt with a loss of less than 1% between 50 and 5 km of depth and then rapidly increases to 6% by a depth of 2 km. Although the trends in carbon vaporization with depth are different in the numerical models compared to our atomistic simulations, both models predict that carbon is completely devolatilized before reaching the surface, while hydrogen is mostly retained in the melt.

A recent study using Fe K-edge x-ray absorption near-edge structure spectroscopy combined with thermodynamic calculations at 2173 K find that the speciation of the atmosphere created by a magma ocean is highly sensitive to the oxygen fugacity and volatile proportions (*36*). At an oxygen fugacity of ΔIW + 0.5 and a temperature of 2173 K, the resulting atmosphere above a magma ocean with 0.01 wt % C and 0.01 wt % H is dominated by CO with a total atmospheric pressure of 0.14 kbar and C/H ratio of 4.5. Thermochemical equilibrium calculations on gases released by a bulk silicate Earth composition at a pressure of 0.1 kbar and temperatures of 500 to 4000 K demonstrate that the relative proportion of volatiles in the gas phase are strongly influenced by temperature, total concentration of carbon and hydrogen, and oxygen fugacity (*28*). At 4000 K and 0.1 kbar, the C/H ratio of the gas was found to be 0.2 for approximately nominal C and H abundances (0.006 wt % C and 0.006 wt % H) (*28*). Using the vaporization proportions calculated in this study at 4000 K and 1 kbar (10× the pressure of the thermochemical equilibrium calculations), we would predict a gas C/H ratio of ~3 for nominal C and H abundances, assuming that the vaporization trends still hold at lower volatile abundances. The volatility of hydrogen depends strongly on temperature and likely changes very rapidly between 0.1 and 1 kbar, which could change the C/H ratio by an order of magnitude.

Thermodynamic calculations of volcanic gases forming from MORB melt with 1000 parts per million (ppm) H_2_O (0.006 wt % H) and 500 ppm CO_2_ (0.02 wt % C) at 1573 K predict that the proportion of hydrogen in the gas changes rapidly between pressures of 0.01 and 1 kbar (*34*). While at 1 kbar, the volcanic gas consists almost entirely of CO_2_ with less than 1% H_2_O, the proportion of H_2_O relative to CO_2_ grows to 30% by 0.1 kbar and 75% by 0.01 kbar. A similar rapid drop in H_2_O content with increasing pressure was predicted for basalts with 1100 to 7000 ppm H_2_O and 650 to 1100 ppm CO_2_ in a similar more recent study (*37*). A recent numerical model based on mixing length theory on a bridgmanite melt with 120 ppm CO_2_ (0.003 wt % C) and 410 ppm H_2_O (0.002 wt % H) proposes that the early atmosphere of Earth was dominated by CO_2_ and depleted in H_2_O during the existence of a global magma ocean with a resulting CO_2_ partial pressure of 0.1 to 1 kbar, depending on the initial conditions and global melt fraction (*35*). The volatile vaporization results from our ab initio calculations of volatile-rich pyrolite melt, particularly the prediction of a CO_2_-rich and H_2_O-depleted atmosphere, generally agrees with the numerical models of the basaltic melts and the mixing length theory calculations on bridgmanite melt.

We predict that in the early stages of the magma ocean, covered by a thick and hot atmosphere, the magma ocean released significantly more carbon than hydrogen, especially at depths approaching the surface. It is only after the pressure of the atmosphere decreased that much of the hydrogen devolatilized from the pyrolite melt, inversing the flux of carbon and hydrogen to the atmosphere. Eventually, water was the main volatile species released from the magma ocean. If this happened after the separation of a shallow magma ocean and a basal magma ocean (*20*, *38*), then a significant portion of hydrogen was conserved in the molten silicate as hydroxyls, which would eventually be stored in hydrous minerals after the crystallization of the magma ocean (*39*).

The presence of a thick atmosphere has been shown to slow down the cooling rate of global magma oceans (*15*–*17*). Because of the greenhouse nature of CO_2_ and H_2_O absorbing and emitting radiation within the thermal infrared range, their concentration in the atmosphere will have a considerable effect on the cooling rate of early Earth, as it would enhance the thermal insulating character of the atmosphere. While CO_2_ and H_2_O have a strong greenhouse effect, CO has a weak greenhouse effect, and H_2_ has no greenhouse effect (*40*); thus, quantifying their relative proportions in the atmosphere is important for understanding the cooling history of very early Earth. In this study, we have shown that the relative proportion of CO_2_ increases with increasing oxidation state, decreasing density, and decreasing temperature, which suggests that the abundance of CO_2_ increased with time, effectively prolonging the cooling rate of early Earth’s global magma ocean. The total amount of hydrogen and carbon and their oxidation states after the giant impact are still open questions today that require further research.

## METHODS

We perform first-principles molecular dynamics simulations with the projector-augmented wave method (*41*) of density functional theory using the Vienna Ab initio Simulation Package (*42*). We treat the electron exchange and correlation in the Perdew-Burke-Ernzerhof form (*43*) of the generalized gradient approximation. The kinetic energy cutoffs for the plane-wave expansion of the wave functions and the augmentation charges are set to 550 and 800 eV, respectively. We use the NVT canonical ensemble, where the number of atoms (N) and the volume (V) of the simulation box are fixed, and the temperature (T) are controlled with a Nosé-Hoover thermostat (*44*). The simulations are performed over a grid of volumes at each temperature and generally last 10 to 50 ps with a time step of 0.5 to 1 fs, depending on the density, temperature, and composition (e.g., longer durations at low temperatures and shorter time steps with the presence of hydrogen). Each time step corresponds to a snapshot, and all snapshots are taken into account for calculating the speciation statistics. The size of the simulation cell is large enough such that the Brillouin zone can be sampled at the Gamma point. The mean square displacement as a function of time shows a ballistic regime below approximately 100 fs, after which the atoms reach a diffusive regime.

The composition of bulk silicate Earth is modeled with a pyrolite (*23*, *20*) melt with the stoichiometry, NaCa_2_Fe_4_Mg_30_Al_3_Si_24_O_89_ (table S1). We add carbon and hydrogen in various forms and amounts: 4CO (3 wt % CO), 8CO (5 wt % CO), 4CO_2_ (6 wt % CO_2_), 8CO_2_ (10 wt % CO_2_), 4H_2_O (2 wt % H_2_O), and 8H_2_O (4 wt % H_2_O) formula units to the 153-atom pyrolite supercell (see table S2 for more detailed volatile concentrations). We conduct simulations at five temperatures (3000, 4000, 5000, 6000, and 7000 K) for pyrolite + 3 wt % CO and pyrolite + 5 wt % CO_2_ and at 4000 K for pyrolite + 6 wt % CO and pyrolite + 10 wt % CO_2_ (see table S3 for full summary of the calculations performed in this study). In addition, we add hydrogen to the pyrolite + 3 wt % CO supercells to examine the effect of hydrogen on the volatility of carbon through the substitution 2 Mg + 1Si ↔ 8H (3 wt % CO + 0.25 wt % H), resulting in a minimal change of the Mg/Si ratio and oxidation state. To determine the volatility of carbon in a relatively carbon-poor melt, we build a supercell with 1CO unit, resulting in a melt with 0.9 wt % CO (0.4 wt % C). Because of low abundance of carbon (1 atom per simulation), we perform the simulation for ~30,000 fs and remove spin polarization. To examine the volatility behavior of hydrogen in water-saturated pyrolite melts, we simulate pyrolite with 10H_2_O and 10H_2_ (10 wt % H_2_O) and pyrolite with 20H_2_O (10 wt % H_2_O) at 4000 K, representing relatively more reduced and more oxidized melts, respectively.

We consider densities ranging from ~2.7 g/cm^3^ (corresponding to a pyrolite melt with no carbon-bearing nanobubbles) to an average melt-vapor density of ~0.95 g/cm^3^. This range is broad enough to allow us to observe the formation of nanobubbles and examining the gas-like volatile species that are vaporized within those bubbles. Lower densities become progressively more difficult to achieve because of increasing computational costs. Over the course of the simulation, volatile species are able to continuously transverse between the void space as vapor molecules and the interconnected silicate melt as dissolved volatile species, where the predisposition to exist in the vapor phase over the melt phase depends primarily on the pressure and temperature of the system. The location, size, and shape of the bubbles continuously change over the course of the simulation. See fig. S6 for example trajectories of carbon over the course of the simulation. We test the effect of doubling the 150-atom supercell on the volatility of carbon at ~0.95 g/cm^3^ and 5000 K, resulting in nearly identical vaporization values and CO/CO_2_ ratios (within 2%). The d-electrons of iron are treated as spin-polarized at all temperatures, pressures, and compositions for the ~150-atom supercell. For the ~300-atom supercell, we perform non–spin-polarized calculations due to computational costs, which do not affect the behavior of carbon.

Bond distances were determined from the pair distribution functions (PDFs), which describe the probability of finding an atom type at a given distance from the reference atom. The first coordination sphere of atoms that are directly bonded to the reference atom appear as the first peak in the PDFs, and so, the first minimum of the PDFs becomes the bond criterion between two atom types. For example, at 4000 K, we calculate an average C─O bond length of ~1.2 Å with a distribution of approximately ±0.7 Å, resulting in a maximum acceptable bond length of ~1.9 Å. Cation-oxygen bond lengths were roughly constant with density within the pressure range examined in this study; thus, bond lengths were averaged over densities at each temperature. We calculate the volatility and speciation of carbon and hydrogen in the pyrolite melts with the speciation module of the Universal Molecular Dynamics package (*45*). Vaporized species are defined as species not bound to the interconnected silicate melt polymer. For example, at one moment for pyrolite + 3 wt % CO, the resulting species might be CO, CO_2_, and NaCa_2_Fe_4_Mg_30_Al_3_Si_24_C_2_O_90_, indicating that half of the carbon exists as vapor species (CO and CO_2_), while the other half is bonded to the silicate melt (NaCa_2_Fe_4_Mg_30_Al_3_Si_24_C_2_O_90_). The proportion of species in the vapor and melt phases is then calculated from an average over the entire simulation.
