# Supplementary material for: Genesis of a CO2-rich and H2O-depleted atmosphere from Earth’s early global magma ocean
Source: Sci Adv. 2021 Oct 6;7(41):eabj0406. doi: 10.1126/sciadv.abj0406 (PMC8494444; doi:10.1126/sciadv.abj0406)
Supplement: Supplementary file 1 — Figs. S1 to S6 Tables S1 to S3 [file sciadv.abj0406_sm.pdf]

Science Advances
AAAS

Supplementary Materials for

Genesis of a CO_2-rich and H_2O-depleted atmosphere from Earth's early global magma ocean

Natalia V. Solomatova* and Razvan Caracas

*Corresponding author. Email: natalia.solomatova@ens-lyon.org

Published 6 October 2021, *Sci. Adv.* **7**, eabj0406 (2021)
DOI: 10.1126/sciadv.abj0406

This PDF file includes:

Figs. S1 to S6
Tables S1 to S3

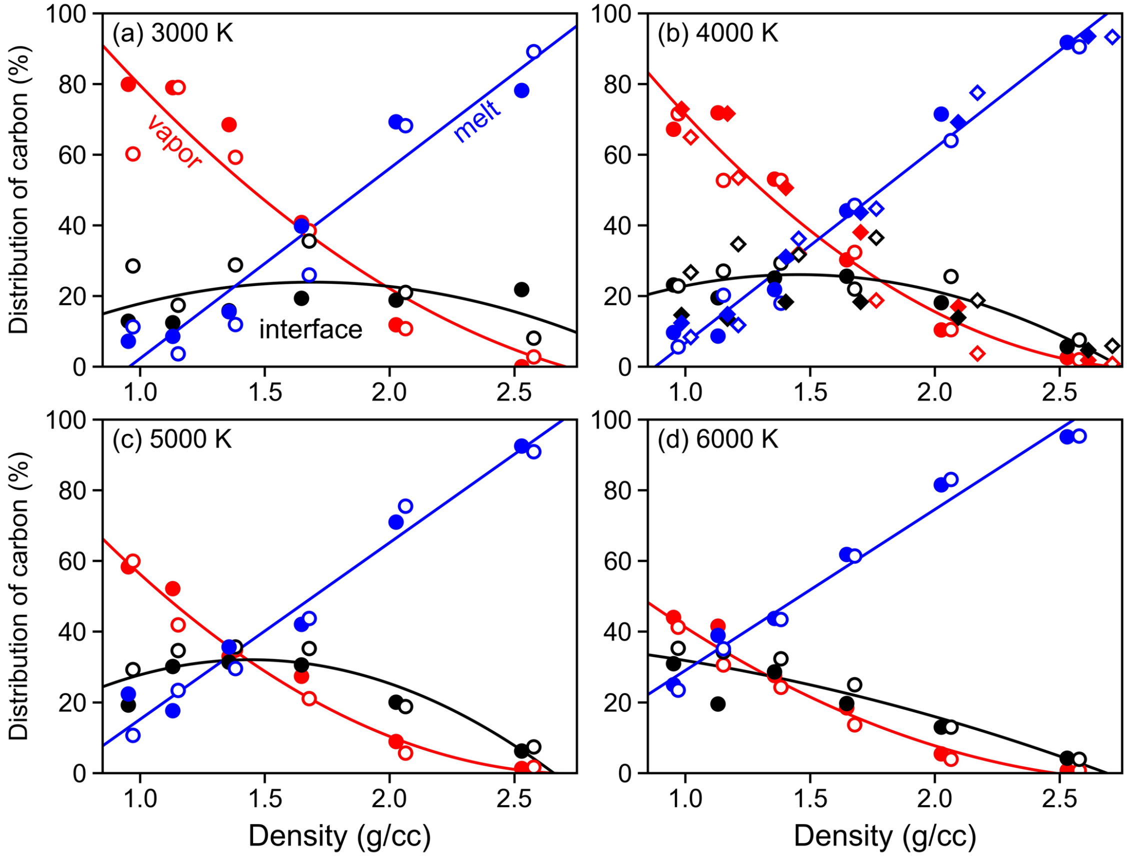

Fig. S1. Proportion of carbon in the vapor of the bubbles (red), attached to the bubble interface (black) and deep in the silicate melt (blue). Filled circles are pyrolite + 3 wt% CO, open circles are pyrolite + 5 wt% CO_2, filled diamonds are pyrolite + 6 wt% CO, and open diamonds are pyrolite + 10 wt% CO_2.

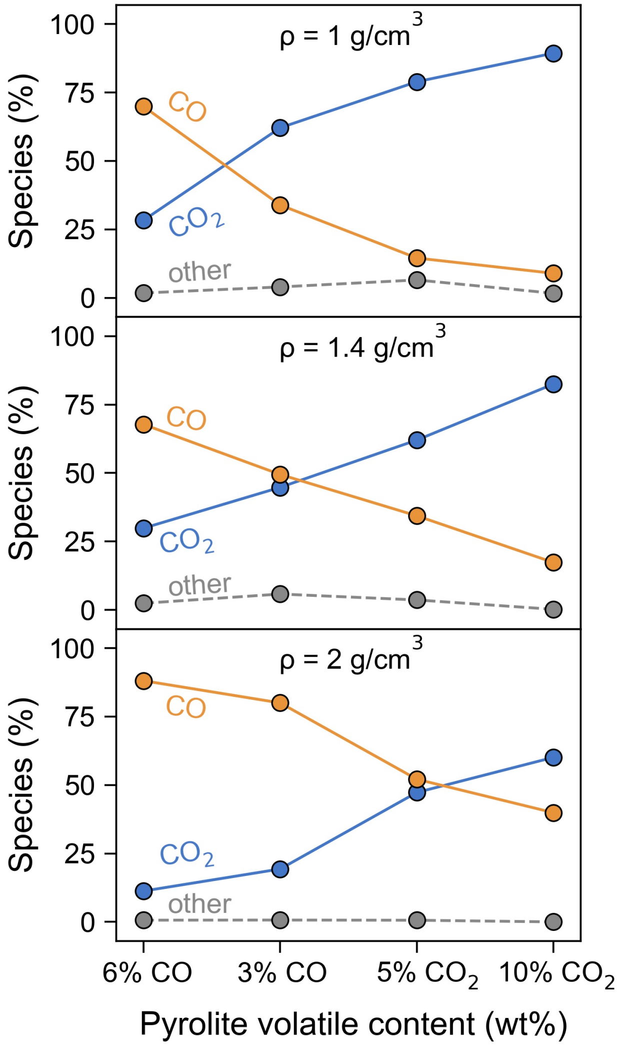

Fig. S2. Relative proportion of CO (orange), CO_2 (blue) and other species (grey) in the vapor phase as a function of volatile content and type at densities of approximately 1, 1.4 and 2 g/cm^3. The content of CO and CO_2 in the pyrolite system can be used as an indirect marker for the oxygen fugacity. Species labeled as "other" include vaporized silicate species, such as SiO, SiO_2, O and O_2 (see Fig. S5 for their relative abundances in the vapor phase).

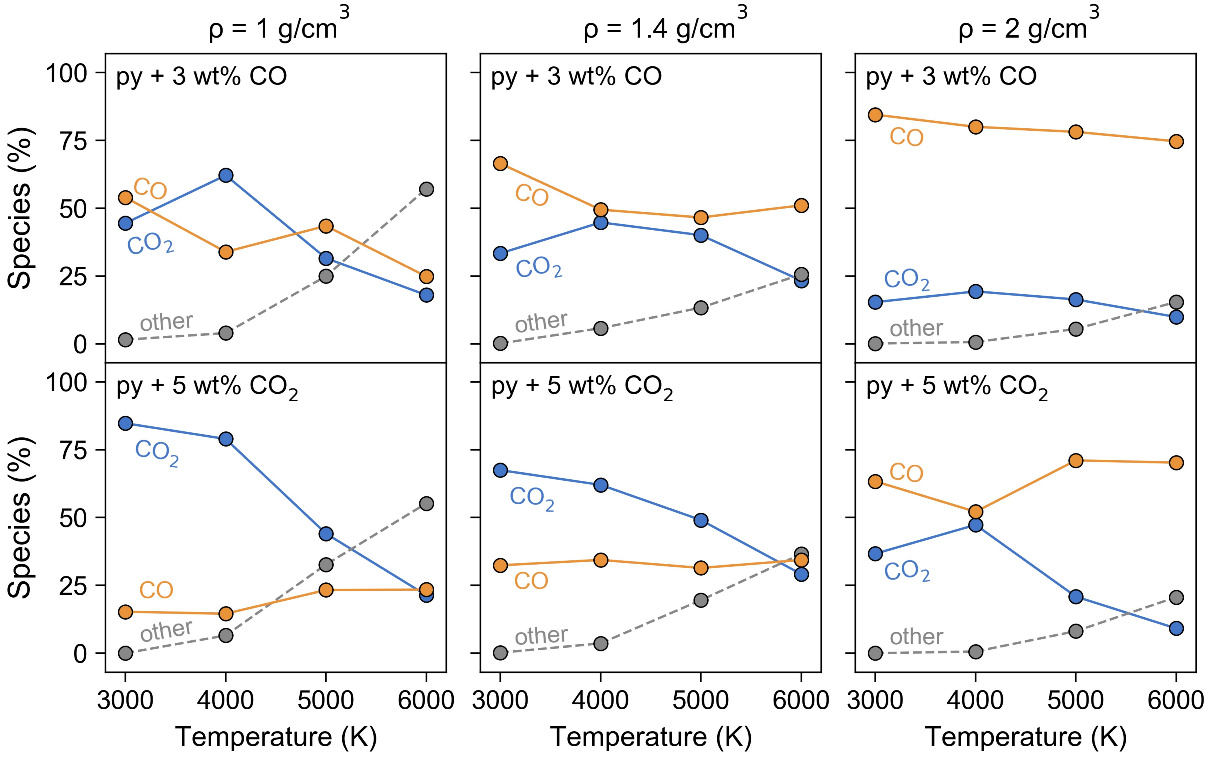

Fig. S3. Relative proportion of CO (orange), CO_2 (blue) and other species (grey) in the vapor phase as a function of temperature for pyrolite ("py") with 3 wt% CO and 5 wt% CO_2 at densities of approximately 1, 1.4 and 2 g/cm^3. Species labeled as "other" include vaporized silicate species, such as SiO, SiO_2, O and O_2 (see Fig. S5 for their relative abundances in the vapor phase).

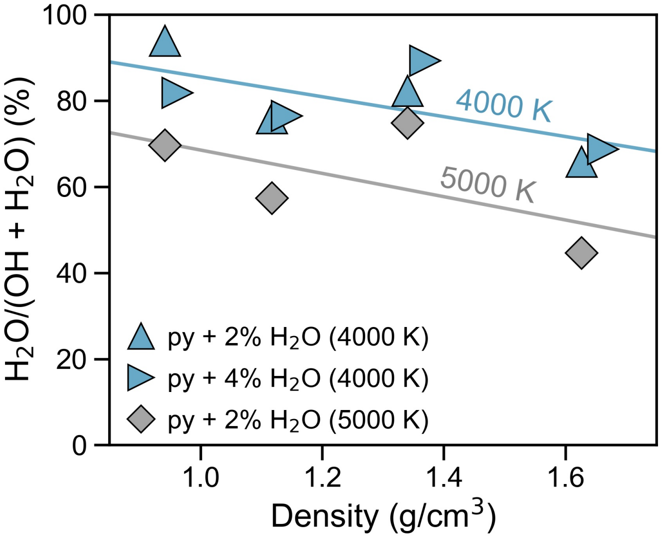

Fig. S4. Relative proportion of H_2O relative to OH^- in the vapor phase in pyrolite with 2-4 wt% H_2O at 4000 K (blue) and 5000 K (grey). Proportions at 3000 K are not shown due to the near absence of hydrogen in the vapor phase (see Fig. 2 of the main text). Linear fits at 4000 K and 5000 K are guides for the eyes, showing a general decrease of H_2O relative to OH^- with increasing density.

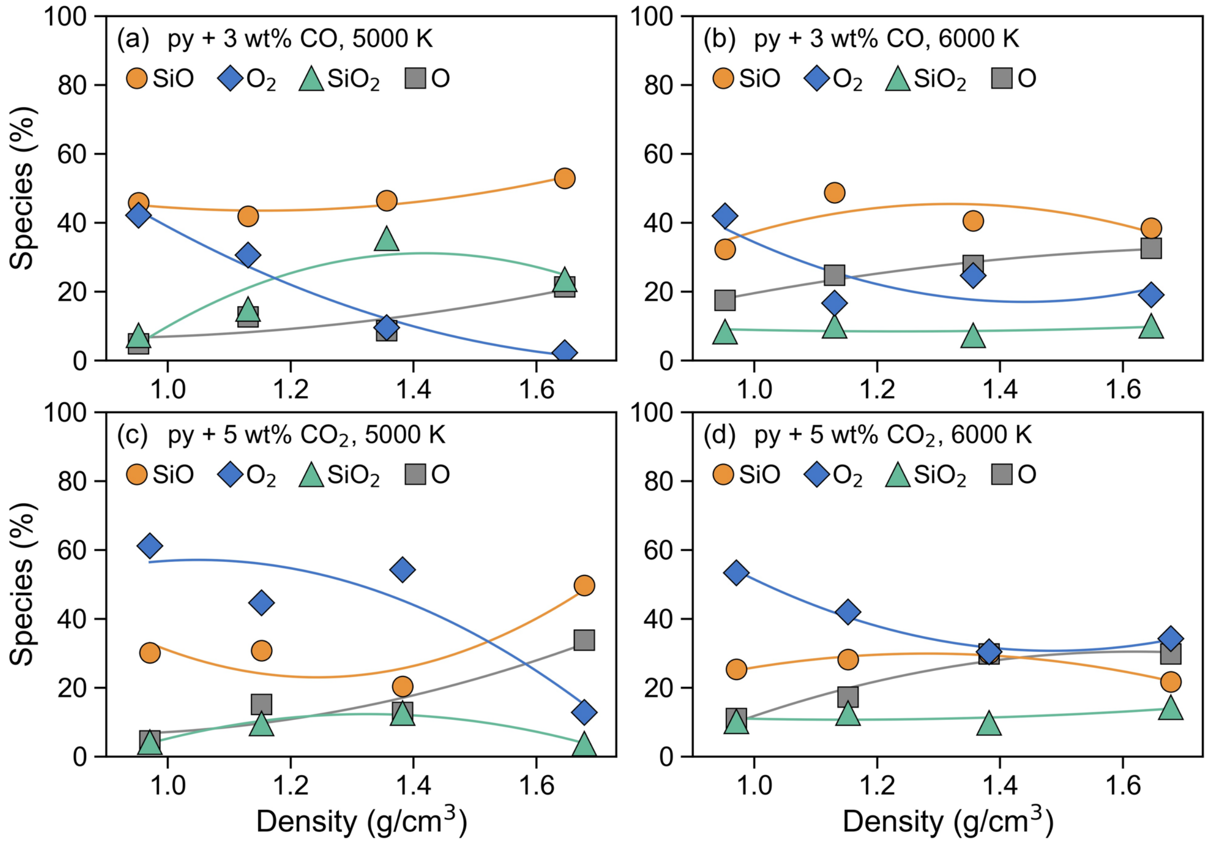

Fig. S5. Relative proportion of SiO, SiO_2, O and O_2 in the vapor phase in (a) pyrolite + 3 wt% CO at 5000 K, (b) pyrolite + 3 wt% CO at 6000 K, (c) pyrolite + 5 wt% CO_2 at 5000 K and (d) pyrolite + 5 wt% CO_2 at 6000 K. Polynomial fits are guides for the eyes.

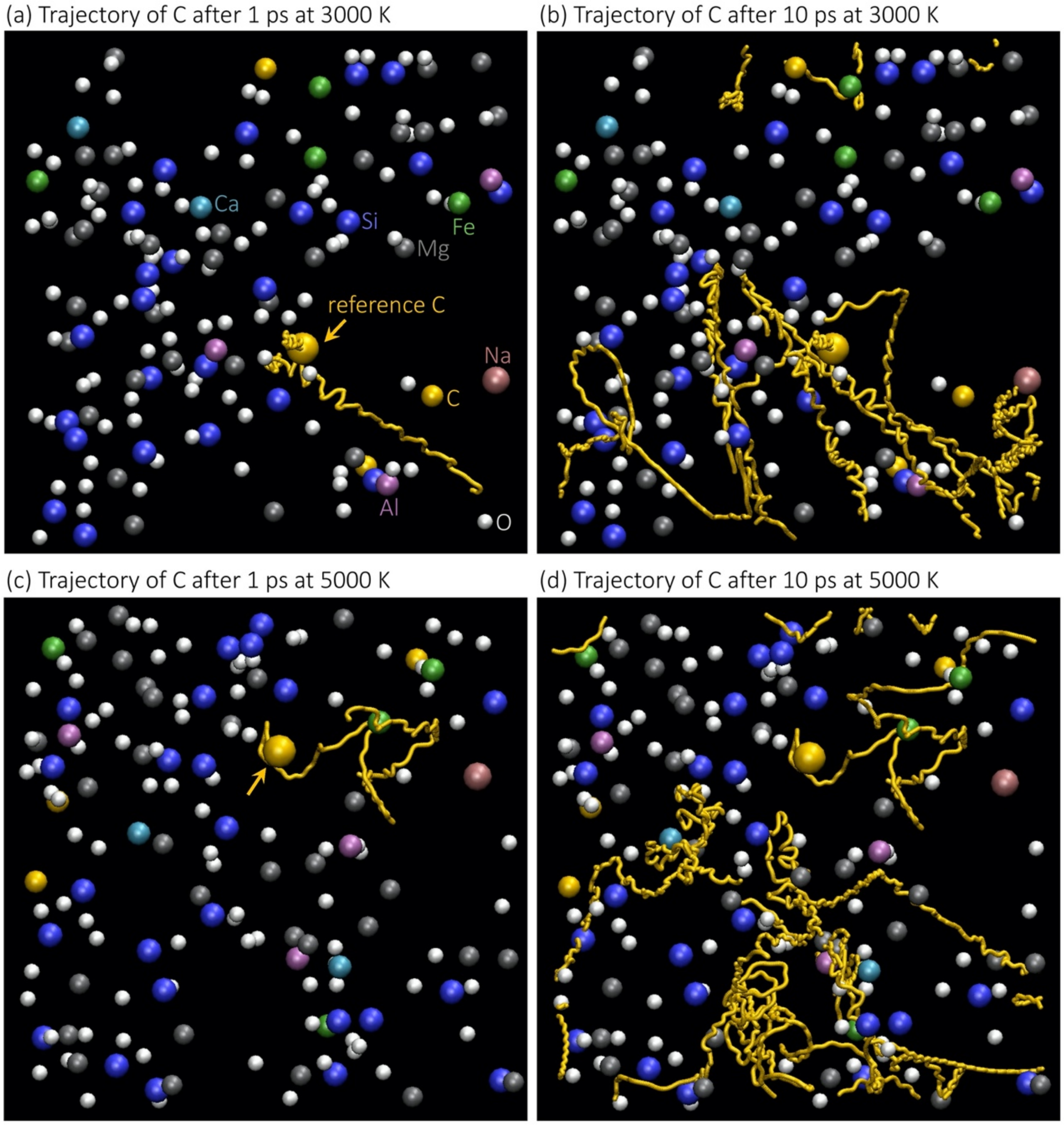

Fig. S6. Example trajectories of a carbon atom in pyrolite melt with 3 wt% CO at about 1.35 g/cm^3 (a) after 1 picosecond at 3000 K, (b) after 10 picoseconds at 3000 K, (c) after 1 picosecond at 5000 K and (d) after 10 picoseconds at 5000 K. Within the first 10 picoseconds, the reference carbon atom traverses between the melt and vapor phase at both temperatures. The duration of the simulations in this study were 10-50 ps, depending on the density, temperature and composition (e.g., longer durations at lower temperatures and lower carbon concentrations).

Table S1. Volatile-free composition of pyrolite in oxide weight percent for the composition of this study and the pyrolite model of McDonough and Sun (1995). The pyrolite model of McDonough and Sun (1995) also includes 0.201 wt.% TiO_2, 0.384 wt.% Cr_2O_3, 0.135 wt. % MnO, 0.25 wt.% NiO, 0.029 wt.% K_2O and 0.021 wt.% P_2O_5, which were not included in our simulations.

	SiO_2	MgO	FeO	Al_2O_3	CaO	Na_2O
This study	44.6	37.4	8.88	4.73	3.47	0.96
Pyrolite model	45.0	37.8	8.05	4.45	3.55	0.36

Table S2. Carbon and hydrogen concentrations in pyrolite melt reported as elemental weight percent, elemental mole percent and oxide weight percent. The melt name includes the number of formula units added to the volatile-free pyrolite melt, $NaCa_2Fe_4Mg_{30}Al_3Si_{24}O_{89}$. Here we tabulate the carbon concentrations both as wt% CO and wt% CO_2 for all carbon-bearing compositions. In the main text, we report the concentrations as they were added to the melt (e.g., py+4CO is pyrolite + 3 wt% CO and py+4CO_2 is pyrolite + 5 wt% CO_2).

Melt name	wt% C	mol% C	wt% CO	wt% CO_2	wt% H	mol% H	wt% H_2O
py+4CO	1.44	2.48	3.35	5.26	0	0	0
py+4CO_2	1.41	2.42	3.28	5.16	0	0	0
py+8CO	2.78	4.73	6.48	10.2	0	0	0
py+8CO_2	2.68	4.52	6.25	9.82	0	0	0
py+4H_2O	0	0	0	0	0.24	4.85	2.18
py+8H_2O	0	0	0	0	0.48	9.04	4.27
py+4CO+8H	1.47	2.41	3.42	5.37	0.25	4.82	2.20
py+10H_2O+10H_2	0	0	0	0	1.17	19.7	10.5
py+20H_2O	0	0	0	0	1.12	18.8	10.0
py+1CO	0.37	0.65	0.86	1.35	0	0	0

Table S3. Summary of the simulations that were performed in this study.

Melt name	Temperature range (K)	Density range (g/cm^3)
py+4CO	3000 - 7000	0.95 - 2.53
py+4CO_2	3000 - 7000	0.97 - 2.58
py+8CO	4000	0.99 - 2.61
py+8CO_2	4000	1.02 - 2.71
py+4H_2O	3000 - 5000	0.94 - 2.50
py+8H_2O	4000	0.96 - 2.55
py+4CO+8H	4000	0.93 - 2.48
py+10H_2O+10H_2	4000	1.39
py+20H_2O	4000	1.22
py+1CO	4000	1.32
